# NOACs Added to WHO’s Essential Medicines List: Recommendations for Future Policy Actions

**DOI:** 10.5334/gh.774

**Published:** 2020-10-06

**Authors:** Mariachiara Di Cesare, Jordan D. Jarvis, Oana Scarlatescu, Xinyi Leng, Ezequiel J. Zaidel, Esteban Burrone, Jean-Luc Eiselé, Dorairaj Prabhakaran, Karen Sliwa

**Affiliations:** 1 Middlesex University, London, GB; 2 London School of Hygiene and Tropical Medicine, London, GB; 3 World Heart Federation, Geneva, CH; 4 The Chinese University of Hong Kong, Shatin, HK; 5 Cardiology Department, Sanatorio Güemes, and the University of Buenos Aires, Buenos Aires, AR; 6 Medicines Patent Pool, Geneva, CH; 7 Public Health Foundation of India, Gurgaon, IN; 8 Hatter Institute for Cardiovascular Research in Africa, University of Cape Town, ZA; 9 CHI, Faculty of Health Sciences, University of Cape Town, ZA

**Keywords:** WHO essential medicines list, NOACs, access to essential medicines, atrial fibrillation

## Abstract

The introduction of non-vitamin K antagonists oral anticoagulants, a class of medicines which includes dabigatran, apixaban, edoxaban and rivaroxaban, has resulted in improvements in the safety and efficacy of non valvular atrial fibrillation treatment for stroke prevention, with significant reductions in stroke, intracranial haemorrhage, and mortality. For these reasons, a team of World Heart Federation Emerging Leaders led efforts to add non-vitamin K antagonists oral anticoagulants to the World Health Organization’s Model List of Essential Medicines in 2019. Following the inclusion of this class of medicines in the Essential Medicines List, this editorial proposes several recommendations to improve the accessibility, affordability and acceptability of non-vitamin K oral anticoagulants, especially in low- and middle-income settings, in order to successfully manage non-valvular atrial fibrillation and to lower the risk of stroke.

This editorial aims to inform policy development to increase the availability, affordability and acceptability of non-vitamin K antagonists oral anticoagulant (NOACs), for instance by adding NOACs into the National Essential Medicines lists, following the addition of this class of medicines to WHO Model List of Essential Medicines (EML). It includes the perspectives of several key constituencies, including academia, policy and advocacy, and the World Heart Federation’s leadership, and considers relevant COVID-19 developments for NOACs and cardiovascular health.

Atrial fibrillation (AF) is associated with a higher risk of hospital admissions, cerebrovascular and cardiovascular conditions, and premature death. Over 37.5 million people are affected by AF [1], with 58% of those suffering from it aged 70 years and over. Current projections suggest an expected increase in the incidence and prevalence in the next three decades due to population growth, ageing, and better survival among patients with AF [2,3,4]. By 2020, an estimated 17.9 million people in low- and middle-income countries (LMICs) will be living with nonvalvular atrial fibrillation (NVAF) [5]. Many of those affected by AF in LMICs are, less likely to access preventive measures and to receive a timely diagnosis (e.g., lack of access to ECG) and the anti-coagulation treatment they require as per AF management guidelines (see Table 1), which leads to significant health and economic burden [6,7,8].

**Table 1:** Treatment guidelines by stroke risk stratification for patients with AF.

	Low stroke risk (CHADSVASC = 0)	Moderate stroke risk (CHADSVASC = 1)	High stroke risk (CHADSVASC ≥ 2)

AHA/ACC	No anticoagulants	OACs or aspirin (LOE IIb)	NOACs (dabigatran, rivaroxaban, apixaban, and edoxaban) are recommended over warfarin (IA)
ESC	No antiplatelet or anticoagulant treatment (IIIB)	OAC should be considered (IIaB)	NOAC (IA)c, VKA (IA)c,d
NICE	No antithrombotic therapy	Consider OAC (Warfarin or NOACs Apixaban, Dabigatran, Rivaroxaban)	Offer OAC (Warfarin or NOACs Apixaban, Dabigatran, Rivaroxaban)
ASIA PACIFIC	No anticoagulants	OACs	NOACs preferred to warfarin
CANADA	No antithrombotic therapy	OAC or Aspirin	NOACs
AUSTRALIA	No anticoagulants	OACs (GRADE: Strong; Evidence: Moderate).	Warfarin, NOAC (apixaban, dabigatran or rivaroxaban) (GRADE: Strong; Evidence:High.)
ARGENTINA	No anticoagulants	OACs or aspirin (LOE I B)	OAC (IA) with Warfarin or NOACs
MEXICO			Dabigatran or Warfarin (Recommended 2011)
BRAZIL	No anticoagulants	OAC (LOE IIaC)	OAC (IA) with Warfarin or NOACs

Vitamin K Antagonist (VKAs) oral anticoagulants are effective in preventing AF-related strokes [9]; however, patients treated with VKAs must be carefully monitored to effectively reduce the risk of embolic stroke while minimizing bleeding risks. In fact, factors like illness, consumption of vitamin K-rich foods, alcohol, and medications can interfere with patients’ ability to stay within the therapeutic range. Furthermore, the regular monitoring required for VKA treatment is particularly challenging for patients and health systems, especially in remote and resource-limited settings where health systems are often poorly equipped to care for people affected by AF. This regular monitoring can be even more challenging during public health crises such as the COVID-19 pandemic, which led to a decrease in the volume of cardiology visits around the world [10,11,12].

In the past ten years, the introduction of NOACs, a class of medicines which includes dabigatran, apixaban, edoxaban and rivaroxaban [13], has resulted in improvements in the safety and efficacy of NVAF treatment for stroke prevention, with significant reductions in stroke, intracranial haemorrhage, and mortality [14]. The advantage of NOACs over VKAs is that patients on NOACs do not require routine monitoring and are not subject to the same treatment interference challenges such as the risk of bleeding, drug-drug interactions, dietary restrictions. Accordingly, major clinical practice guidelines worldwide recommend NOACs over VKAs for initial treatment of NVAF for stroke prevention [14]. Unfortunately, NOACs are less available and affordable in LMICs where they are most needed [15].

For these reasons, a team of World Heart Federation Emerging Leaders led efforts to add NOACs to the WHO Model List of Essential Medicines (EML). The team involves experts in cardiology, neurology and public health from ten countries which concluded, in their successful appeal to WHO, that NOACs were not only safer (lower risk of major bleeding) and more effective, but also more cost-effective than VKAs in preventing stroke and systemic embolism in NVAF patients [14]. These conclusions were based on findings from pivotal randomized controlled trials [16,17,18,19] and large-scale, real-world registries [20,21,22]. This new application overcame the limitations of a previous attempt [23] to add NOACS to the EML which was rejected for several reasons, including the belief that evidence based only on trial populations would not be representative of patients who would receive such treatment in real-world practice, the lack of specific antidotes at the time to reverse anti-coagulation, and the higher costs compared to warfarin and to the overall benefits to patients [24,25,26].

The designation as an essential medicine by the WHO signifies the therapeutic value and importance of NOACs. The WHO EML listing is meant to lead to a cascade of actions that will improve equitable access to essential medicines, considering that many governments utilize the WHO EML as a model for setting their own medicine priorities [27]. Bringing NOACs within reach of those who need them most, especially in low- and middle-income countries, is the next frontier in ensuring access to anti-coagulation therapy, successfully managing NVAF and lowering the risk of stroke. To achieve this, we recommend the following policy actions.

## Inclusion of NOACs within Key Health Systems Policies

Making quality NOACs available, affordable, acceptable and accessible in-country will often require governments to include them in their national essential medicines list, clinical treatment guidelines, and other policy tools that ensure their supply and provision in health facilities and pharmacies.

According to data from 2017, 14 countries, primarily in the WHO Europe Region, had listed dabigatran on their national essential medicines lists prior to the WHO recommendation and a subset of those countries also listed apixaban and/or rivaroxaban [28]. This number has already and is expected to further increase after the WHO listing, as medicines listed by WHO tend to be included by a greater number of national EMLs over time, especially in lower-middle and low-income countries [28]. While medicines listed on national EMLs generally become more available and affordable than other medicines, a multitude of diverse factors likely affect priority-setting for national EMLs and various policy and health system barriers must be overcome to ensure equitable access to essential medicines [29,30]. For example, pricing of medicines influences EML inclusion; low-income countries with poor government control on prices appear to be less likely to adopt the WHO EML if the drug prices are deemed too high [31].

Work is also required to increase the acceptability of NOACs, for example, by including it in national guidelines for the management of NVAF and in training packages for healthcare providers. Alignment of policies such as clinical treatment guidelines and EMLs at national and subnational levels can reduce unnecessary policy barriers to care.

## Improving Affordability

In the case of NOACs, the cost is likely one important barrier. Although it is deemed cost-effective, all four NOACs—dabigatran, rivaroxaban, apixaban and edoxaban—remain patent protected in several countries,^1^ and their cost can vary widely. This variation in cost can hinder availability and make NOACs prohibitive in some low-income settings. For example, dabigatran costs USD$65 in the UK and USD$222 in China and rivaroxaban costs approximately USD$60 per patient per month in Kenya [14,32]. Yet factoring in costs associated with VKAs treatment — medication (available at a price as low as USD$1 per month) and health care system costs, such as monitoring requirements — suggests that NOACs are expected to have a lower cost over time [32].

We can learn from actions that have resulted in improvements in global access to expensive, patented HIV and hepatitis C treatments, following their additions to the WHO EML in 2001 and 2015, respectively. These actions include the promotion of generic competition, voluntary licensing of products such as through the Medicines Patent Pool (MPP), and invoking flexibilities enshrined in the World Trade Organization’s Trade Related Aspects of Intellectual Property Rights (TRIPS) agreement [33,34]. MPP reported that generic versions of dabigatran currently exist on the Indian private market, priced as low as between USD$15 and USD$22 per month (the recommended dose of dabigatran is 150 mg twice a day).^2^ Cost of production modelling further indicated that NOACs could be produced at even lower costs, and thus it is expected that with greater volumes and market shares, it could be made available at lower prices while maintaining profitability [32]. Moving forward, it is estimated that licensing NOACs to MPP could allow generic entry years before patent expiry in a large number of LMICs and thereby “facilitate up to [an additional] 1.9 million patient-years of treatment for both nonvalvular atrial fibrillation and venous thromboelism [32].”

In order for NOACs to be affordable to households, governments should consider including them in health insurance packages and other financial schemes, in addition to efforts to lower costs already discussed.

Taken together, these solutions have the potential to accelerate more equitable global access to care and essential medicines for people affected by NVAF and at risk of stroke who may otherwise not benefit from anti-coagulation therapy because of the high monitoring costs of VKAs and the lifestyle challenges of remaining within the therapeutic range. These solutions are also in line with the World Heart Federation’s policy recommendations on ensuring access to medicines for circulatory diseases, including strengthening health systems, designing and implementing creative financing models and investing in the health workforce [35].

## Footnotes

According to https://www.medspal.org/, as of August 2020, there were patents pending or granted on the **dabigatran** compound or its salt form in Argentina, Belarus, Brazil, Chile, China, Colombia, Egypt, India, Indonesia, Kazakhstan, Kyrgyzstan, Malaysia, Mexico, Montenegro, Pakistan, Peru, Philippines, Russian Federation, Serbia, South Africa, Thailand, Turkey, Ukraine, Uzbekistan, Venezuela and Vietnam. There were patents pending or granted on **apixaban** in Albania, Argentina, Brazil, Chile, China, Georgia, India, Indonesia, Kosovo, Kyrgyz Republic, Malaysia, Mexico, Montenegro, North Macedonia, Pakistan, Philippines, Russian Federation, Serbia, South Africa, Thailand, Turkey, Ukraine, Venezuela and Vietnam. **Rivaroxaban** had patents pending or granted in Albania, Argentina, Belarus, Bosnia and Herzegovina, Brazil, Chile, China, Colombia, Cuba, Dominican Republic, El Salvador, Guatemala, Honduras, India, Indonesia, Jamaica, Mexico, Morocco, North Macedonia, Pakistan, Peru, Philippines, Russian Federation, Serbia, South Africa, Trinidad and Tobago, Turkey, Ukraine, Uruguay and Venezuela. **Edoxaban** had patents pending or granted in Argentina, Brazil, China, Egypt, India, Indonesia, Malaysia, Mexico, Pakistan, Philippines, Russian Federation, South Africa, Thailand, Turkey and Vietnam.

The price is as found on www.1mg.com for Dabipla/Goodflo (150mg twice per day for 30 days).
